# Expression of *Helicobacter pylori hspA* Gene in *Lactococcus lactis* NICE System and Experimental Study on Its Immunoreactivity

**DOI:** 10.1155/2015/750932

**Published:** 2015-04-22

**Authors:** Xiao-Juan Zhang, Shu-Ying Feng, Zhi-Tao Li, Yan-Ming Feng

**Affiliations:** ^1^Department of Pathogen Biology, Medical College, Henan University of Science and Technology, Luoyang 471003, China; ^2^Department of Immunology, Medical College, Henan University of Science and Technology, Luoyang 471003, China; ^3^Department of Epidemiology, Medical College, Henan University of Science and Technology, Luoyang 471003, China

## Abstract

*Aim*. The aim of this study was to develop an oral *Lactococcus lactis* (*L. lactis*) vaccine against *Helicobacter pylori* (*H. pylori*). *Methods*. After *L. lactis* NZ3900/pNZ8110-*hspA* was constructed, growth curves were plotted to study whether the growth of recombinant *L. lactis* was affected after *hspA* was cloned into *L. lactis* and whether the growth of empty bacteria, empty plasmid bacteria, and recombinant *L. lactis* was affected by different concentrations of Nisin; SDS-PAGE and Western blot were adopted, respectively, to detect the HspA expressed by recombinant *L. lactis* and its immunoreactivity. *Results*. There was no effect observed from the growth curve after exogenous gene *hspA* was cloned into *L. lactis* NZ3900; different concentrations of Nisin did not affect the growth of NZ3900 and NZ3900/pNZ8110, while different concentrations of Nisin inhibited the growth of NZ3900/pNZ8110-*hspA* except 10 ng/mL Nisin. No HspA strip was observed from SDS-PAGE. Western blot analysis showed that HspA expressed by recombinant bacteria had favorable immunoreactivity. *Conclusion*. The growth of recombinant *L. lactis* was suppressed even though a small amount of HspA had been induced to express. Therefore recombinant *L. lactis* only express HspA which was not suitable to be oral vaccine against *Helicobacter pylori*.

## 1. Introduction

*Helicobacter pylori* (*H. pylori*) is a spiral and motile Gram-negative bacterium, which colonizes the human stomach mucosa and is the main cause of a range of gastric and duodenal diseases including chronic gastritis, stomach and duodenal ulcers, MALT lymphoma, and gastric adenocarcinoma, and it is considered as the second most frequent cause of cancer death worldwide [1, 2].* H. pylori* is also the pathogenic microorganism which has relationship with diabetes mellitus, coronary artery disease, thrombocytopenic purpura, and so on [3–5]. Heat shock proteins (Hsp) of* H. pylori* play important roles in* H. pylori* adhesion to gastric epithelial, assembling of urease enzyme subunit and stimulating the body to produce an immune response; at the same time they are the main immunogens of* H. pylori*. HspA is the main functional unit of Hsp and it locates bacterial surface, is relatively conservative, and has immunogenicity, so it is an important candidate vaccine antigen of* H. pylori*.

*Lactococcus lactis* (*L. lactis*) is a Gram-positive, noninvasive, nonpathogenic, and food-grade bacterium and is generally recognized as safe (GRAS), which has been used in the food industry for production of fermented milk products for many years. Because of its genetic accessibility and easy maneuverability,* L. lactis* has been extensively used as a live delivery vehicle of heterologous proteins [6–8]. Nisin-controlled expression (NICE) system is the most effective food-grade inducible expression system. As induction signal, Nisin has been successfully applied to a variety of strains, including* Lactococcus*,* Lactobacillus*,* Streptococcus*, and* Enterococcus* [9, 10].

To develop an oral vaccine against* H. pylori* in the present study,* hspA* gene was cloned from* H. pylori* MEL-Hp27 and sequenced. Then* hspA* gene was inserted into the vector pNZ8110 and electrotransformed into* L. lactis* NZ3900. The immunoreactivity of HspA expressed by recombinant* Lactococcus* bacteria was identified by western blot analysis.

## 2. Materials and Methods

### 2.1. Microorganism, Plasmid, and Growth Conditions

The strain of* H. pylori* MEL-Hp27 was separated from a clinical atrophic gastritis patient of Zhengzhou city, China.* L. lactis* NZ3900 and the* E. coli*-*L. lactis* shuttle vector pNZ8110 were purchased from NIZO food research.* L. lactis* NZ3900 was grown in M17 medium (Difco, USA) supplemented with 0.5% glucose (GM17) at 30°C. MC1061 was grown in LB broth (Oxoid, UK) at 37°C. When necessary, antibiotics were added to culture medium at the following concentrations: ampicillin (100 *μ*g/mL) and chloramphenicol (10 *μ*g/mL).

### 2.2. PCR of* hspA* Gene

The* hspA* gene was amplified by PCR in the presence of MEL-Hp27 genomic DNA and NcoI-forward primer 5′-catgccatgggcatgaagtttctacca-3′ and XbaI-reverse primer 5′-cggtctagattagtgttttttgtgatc-3′; NcoI/XbaI restriction endonuclease sites were underlined. To construct the translational fusion expression, the four base pairs ATGG of NcoI restriction endonuclease sites under the SD domain of pNZ8110 nisA promoter were added to the forward primer. Two base pairs GC were added to keep the reading frame invariant. PCR was carried out under the following conditions: 30 cycles of denaturation at 94°C for 1 min, annealing at 55°C for 1 min, and extension at 72°C for 1 min.

### 2.3. Construction of the Recombinant NZ3900/pNZ8110-*hspA*

The* hspA* gene was inserted into the* Escherichia coli*- (*E. coli*-)* Lactococcus lactis* (*L. lactis*) shuttle vector pNZ8110 and the recombinant plasmid was transformed into MC1061 using the method of calcium chloride. The transformants were selected on LB plates containing 10 *μ*g/mL chloramphenicol. Identification of the transformants was operated by PCR of the* hspA* gene, digested by NcoI and XbaI, and subjected to DNA sequencing. Then the plasmid of pNZ8110-*hspA* was electrotransformed into* L. lactis* NZ3900 and the transformants were selected on GM17 plates containing 10 *μ*g/mL chloramphenicol.

### 2.4. Influence of* hspA* Gene on the Growth of Recombinant* Lactococcus lactis*

NZ3900 was transferred into the 10 mL GM17 liquid medium according to 1 : 25 (v/v). NZ3900/pNZ8110 and NZ3900/pNZ8110-*hspA* were transferred, respectively, into the 10 mL GM17 liquid medium containing 10 *μ*g/mL chloramphenicol according to 1 : 25 (v/v). Each tube was mixed at interval of 1 hour to measure the absorbance A600 of bacteria. Growth curves of NZ3900, NZ3900/pNZ8110, and NZ3900/pNZ8110-*hspA* were plotted with incubation time as the abscissa and A600 as the vertical axis.

### 2.5. Influence of Nisin Concentration on the Growth of Recombinant* Lactococcus lactis*

NZ3900/pNZ8110-*hspA* was transferred, respectively, into 7 tubes of 10 mL GM17 liquid medium containing 10 *μ*g/mL chloramphenicol according to 1 : 25 (v/v); then Nisin stock solution was added to a final concentration of 0 ng/mL, 10 ng/mL, 20 ng/mL, 40 ng/mL, 60 ng/mL, 80 ng/mL, and 100 ng/mL. Each tube was mixed at interval of 1 hour to measure the absorbance A600 of bacteria. Growth curves of NZ3900/pNZ8110-*hspA* were plotted with incubation time as the abscissa and A600 as the vertical axis. Both NZ3900 and NZ3900/pNZ8110 were operated as controls at the same time.

### 2.6. Expression of HspA Protein in Recombinant* Lactococcus lactis*

NZ3900/pNZ8110-*hspA* was overnight cultured, then 400 *μ*L of which was transferred, respectively, into 2 tubes of 10 mL GM17 liquid medium containing 10 *μ*g/mL chloramphenicol. After NZ3900/pNZ8110-*hspA* was cultured for 4 hours, Nisin stock solution was added, respectively, to the final concentrations 10 ng/mL and 20 ng/mL to induce the expression of HspA. NZ3900/pNZ8110-*hspA* was sonicated after 4 hours and supernatant protein was collected and quantified using Bradford amount. HspA expressed in* Lactococcus lactis* was detected by SDS-PAGE and Tricine SDS-PAGE.

### 2.7. Detection of the Immunoreactivity of HspA Expressed by Recombinant* Lactococcus lactis*

HspA expressed in recombinant* Lactococcus lactis* was electrophoresed on a 15% SDS-PAGE and then electrotransferred to the cellulose nitrate membrane. The membrane was probed with a primary polyclonal mice antiserum (diluted 1 : 50) and the membrane incubated at 37°C for 2 h, washed three times with TBS/Tween-20 (TBST) for 15 min, and then incubated with the horseradish peroxidase-linked secondary antibody (diluted 1 : 1000). Protein detection was visualized using the DAB western blot detection system (Tiangen, China).

## 3. Results

### 3.1. PCR Results of* hspA* Gene from MEL-Hp27

**Figure 1:**
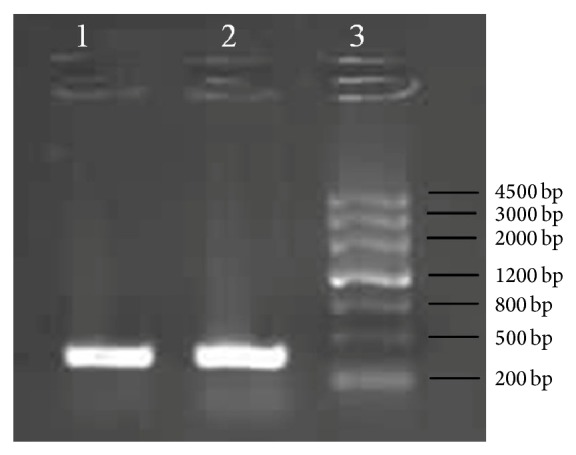
PCR product of MEL-Hp* hspA* gene. (1), (2) PCR product of* hspA* gene from Hp genome; (3) DNA ladder.

Figure 1 showed that the* hspA* gene (Genbank accession number KJ567076) had been cloned successfully, which contained 363 base pairs. The sequencing result showed that the sequences of* hspA* gene in this experiment were correct compared with the sequences of MEL-Hp27* hspA* (Genbank accession number AY295084) except the additional six base pairs, as explained in Section 2.2. The sequence identification result of MEL-HP27* hspA *gene is as follows.

 atgggcatgaagtttc taccattagg agaaagggtc ttagtagaaa gacttgaaga agagaacaaa
 accagttcag gcatcatcat ccctgataac gctaaagaaa agcctttaat gggcgtagtc
 aaagcggtta gccataaaat cagcgagggt tgcaaatgcg ttaaagaagg cgatgtgatc
 gcttttggca aatacaaagg cgcagaaatc gttttagacg gcgttgaata catggtgcta
 gagctagaag acattctagg tattgtgggc tcaggctctt gttgtcatac aaatagtcat
 gaccataaac atgctaaaga gcatgaagct tgctgtcatg atcacaaaaa acactaa

### 3.2. Identification of Recombinant MC1061/pNZ8110-*hspA* and NZ3900/pNZ8110-*hspA*

**Figure 2:**
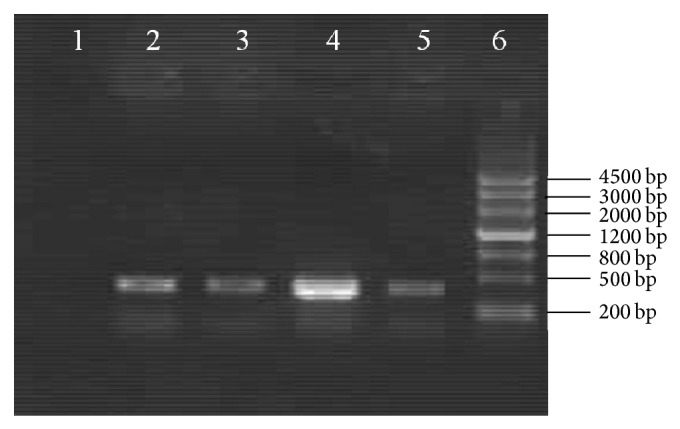
Identification of MC1061/pNZ8110-*hspA* by PCR. (1) Negative control. (2), (3), (4), and (5) PCR product of* hspA* from MC1061/pNZ8110-*hspA*. (6) DNA ladder.

**Figure 3:**
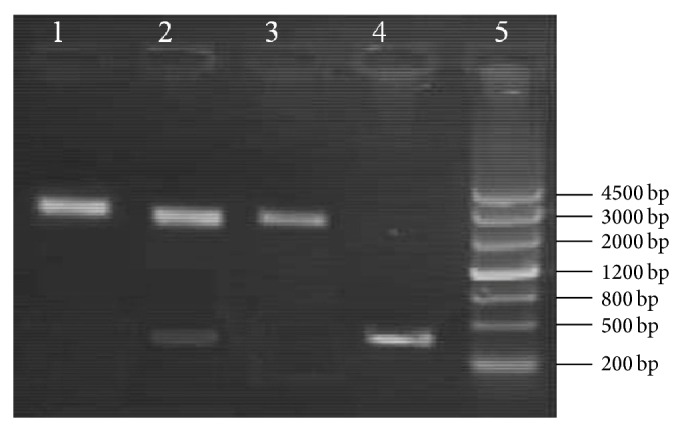
Identification of the recombinant vector of pNZ8110-*hspA* by restriction enzyme digestion. (1) pNZ8110-*hspA* digested by* XbaI*. (2) pNZ8110-*hspA* digested by* NcoI *and* XbaI*. (3) pNZ8110 digested by* XbaI*. (4) PCR product of* hspA*. (5) DNA ladder.

To express the HspA protein in* L. lactis* transformant, the* hspA* gene was inserted into the* E. coli*-*L. lactis* shuttle vector pNZ8110 and the recombinant plasmid was transformed into the* E. coli* MC1061. The recombinant pNZ8110-*hspA* was detected to be recombined properly with PCR (Figure 2) and digestion experiments (Figure 3). Then the recombinant pNZ8110-*hspA* was electrotransformed into* L. lactis* NZ3900. NZ3900/pNZ8110-*hspA* was identified by PCR and digestion experiments (not shown).

### 3.3. Influence of* hspA* Gene on the Growth of Recombinant* Lactococcus lactis*

**Figure 4:**
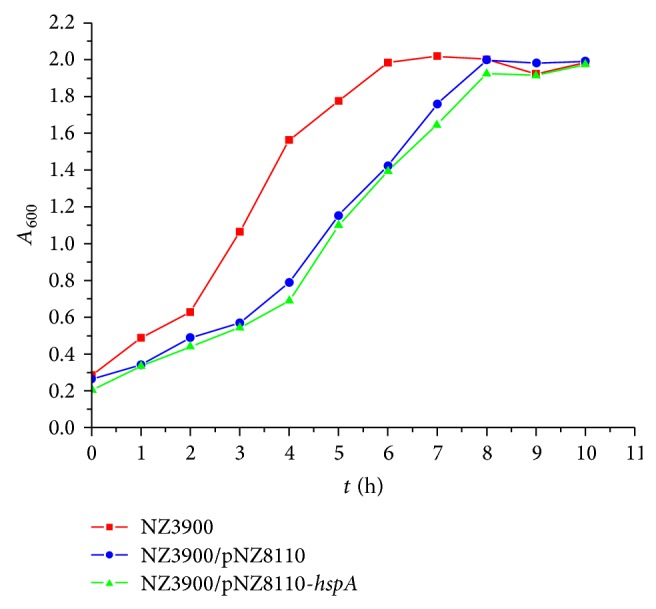
The growth curves of NZ3900, NZ3900/pNZ8110, and NZ3900/pNZ8110*-hspA.*

From the growth curves (Figure 4), it could be seen that the proliferation of NZ3900, NZ3900/pNZ8110, and NZ3900/pNZ8110-*hspA *was very slow within the former 2 hours; then they entered the logarithmic growth phase within the following 6 hours. After about 8 hours, the growth of bacteria got up to the plateau phase and the A600 absorbance value got to 2.0 or so. The proliferation of NZ3900/pNZ8110 and NZ3900/pNZ8110-*hspA* was slower than NZ3900 in the logarithmic growth period, which may be due to the result of plasmid replication, indicating that insertion of* hspA* gene did not impact the growth of recombinant* Lactococcus lactis*.

### 3.4. Influence of Nisin Concentration on the Growth of Recombinant* Lactococcus lactis*

**Figure 5:**
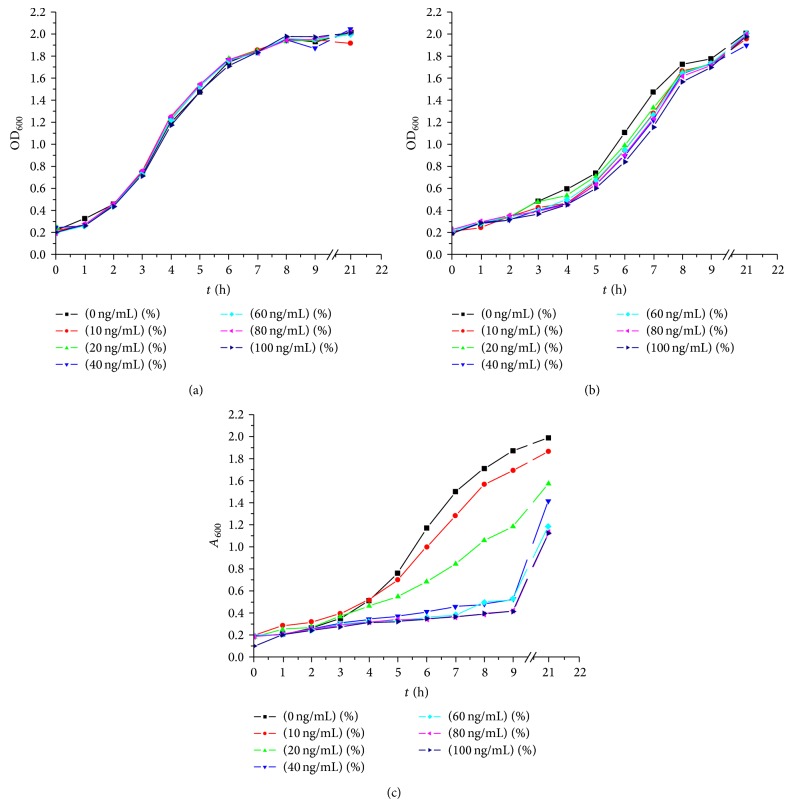
(a) Influence of Nisin concentration on NZ3900. (b) Influence of Nisin concentration on NZ3900/pNZ8110. (c) Influence of Nisin concentration on NZ3900/pNZ8110-*hspA*.

It can be seen from Figures 5(a), 5(b), and 5(c) that the growth of NZ3900/pNZ8110-*hspA* was inhibited noticeably by different concentrations of Nisin. The A600 absorbance value of NZ3900/pNZ8110-*hspA* got to 1.989 in no Nisin group, while in the 100 ng/mL Nisin group it only got to 1.125. The growth curve showed that different concentrations of Nisin did not affect the growth of blank control (NZ3900) and negative control (NZ3900/pNZ8110).

### 3.5. Expression of HspA in Recombinant* Lactococcus lactis*

The expected molecular weight bands could not be observed on the SDS-PAGE gel or Tricine SDS-PAGE gel after 10 ng/mL or 20 ng/mL Nisin induction for 4 hours, indicating that no HspA protein was expressed or the expression level was too low to be detected. The expected molecular weight bands could not be observed in blank control (NZ3900) and negative control (NZ3900/pNZ8110).

### 3.6. Identification of the Immunoreactivity of HspA Expressed by Recombinant* Lactococcus lactis*

**Figure 6:**
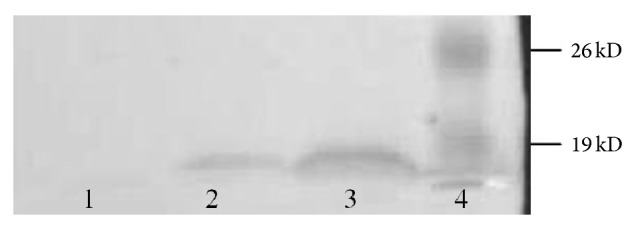
Western blot reaction of HspA protein expressed by* Lactococcus lactis* with polyclonal antibody serum to* H. pylori*. (1) Negative control. (2), (3) Western blot reaction of HspA protein to polyclonal antibody serum to* H. pylori*. (4) Prestained protein marker.

It was presented in Figure 6 that the HspA expressed by* L. lactis* transformant could be recognized by anti-Hp serum. This result indicated that the HspA protein expressed by* L. lactis* transformant had favorable immunoreactivity.

## 4. Discussion

*H. pylori* is now recognized as the most widespread human pathogen. Approximately half of the world's population is infected [11, 12]. A great interest in developing a vaccination method to prevent infection by* H. pylori* has been raised, given that immunization is always considered as the most economic and efficient means in such a field, especially for the developing countries. Many researchers had made a substantial contribution to* H. pylori* vaccine such as protein vaccine or* Salmonella* vector vaccine, while these vaccines could not be optimistical because of the difficult maneuverability or gastrointestinal toxicity or insecurity [13, 14].

*L. lactis* has a number of prominent advantages that make it a promising candidate for large scale gene expression: the bacterium is food-grade, and plasmid selection mechanisms are available that are food-grade and self-cloning. Furthermore, no endotoxins or inclusion bodies are formed and sophisticated genetic tools enable easy genetic handling. Finally, simple, nonaerated fermentation makes direct scale-up from 1-L scale to 1000-L scale possible. The commonly used regulated expression system of* L. lactis* is the Nisin-controlled gene expression (NICE) system [15–18]. In recent years,* L. lactis* has been used to express* H. pylori* antigen protein as live bacteria vehicle. The 3'-region (1152 bp) of the cag7 gene of* Helicobacter pylori *51 strain had been expressed in* Lactococcus lactis *ssp. MG1363 [19]. The results showed that the transformant could produce the Cag7-ct383 protein and the Cag7-ct383 protein level in the* L. lactis* transformant reached a maximum at the early stationary phase without extracellular secretion. The oral administration of the* L. lactis* transformant into mice generated anti-Cag7 antibody in serum in five of five mice. These results suggest that* L. lactis* transformant expressing Cag7-ct383 protein may be applicable as an oral vaccine to induce mucosal and systemic immunity to* H. pylori*.

NICE system was used in our experiments to express the MEL-Hp27 UreB and HspA protein in a food-grade delivery vehicle* L. lactis* NZ3900. The results showed that the maximum yield of the UreB protein was 27.26 *μ*g per milliliter medium and the percentage of UreB to cell extracts of the* L. lactis* transformant got to the peak at 20.19%, which was substantial quantities of antigen expressed in* L. lactis* [20]. In this study, it could be seen that the insertion of* hspA* did not impact the growth of NZ3900/pNZ8110-*hspA* and the bacterial density of NZ3900/pNZ8110-*hspA* could get the same value as NZ3900 and NZ3900/pNZ8110 when they grew into the plateau phase; different concentrations of Nisin did not affect the growth of NZ3900 and NZ3900/pNZ8110, while different concentraions of Nisin significantly inhibited the growth of NZ3900/pNZ8110-*hspA *except 10 ng/mL Nisin; the rest of concentrations of Nisin significantly inhibited the growth of NZ3900/pNZ8110-*hspA*. The expected molecular weight bands could not be observed on the SDS-PAGE gel or Tricine SDS-PAGE gel; western blot identification results showed that HspA expressed by NZ3900/pNZ8110-*hspA* had good immunoreactivity. From the growth curves, it could be inferred that it was not because of Nisin inhibiting the growth of NZ3900/pNZ8110-*hspA* as inhibitory but because HspA had not been induced to be expressed effectively in 10 ng/mL Nisin group, while, in higher concentrations of Nisin group, a small amount of exogenous expression of HspA inhibited the growth of NZ3900/pNZ8110-*hspA* itself. The mechanism deserves to be further investigated.

This study established the experimental foundation for constructing the food-grade vaccine to* H. pylori* infection.
